# Effect of oral L-citrulline on brachial and aortic blood pressure defined by resting status: evidence from randomized controlled trials

**DOI:** 10.1186/s12986-019-0415-y

**Published:** 2019-12-26

**Authors:** Huan-Huan Yang, Xin-Li Li, Wei-Guo Zhang, Arturo Figueroa, Li-Hua Chen, Li-Qiang Qin

**Affiliations:** 1 Department of Nutrition and Food Hygiene, School of Public Health, Soochow University, Suzhou, 215123 China; 2 Freelancer, Irving, TX 75039 USA; 3 Department of Kinesiology and Sport Management, Texas Tech University, Lubbock, TX 79409 USA

**Keywords:** Aortic, Blood pressure, Brachial, L-citrulline, Resting status

## Abstract

**Background:**

Experimental evidence indicates that oral L-citrulline (L-Cit) may reduce resting blood pressure (BP) as well as BP responses to exercise and cold exposure (non-resting). However, results from human intervention trials are inconsistent. This study aims to summarize the clinical evidence regarding the effects of L-Cit supplementation on brachial systolic blood pressure (SBP), brachial diastolic blood pressure (DBP), in addition to aortic SBP and aortic DBP at rest and non-resting conditions.

**Methods:**

Multiple databases including PubMed, Embase, Cochrane library, Web of Science, and Clinical Trials were searched systematically. Randomized controlled trials of human participants were quantitatively meta-analyzed.

**Results:**

Fourteen trials contained in eight studies were available for quantitative syntheses for brachial BP. Results showed that L-Cit supplementation significantly reduced both brachial SBP (− 4.490 mmHg, 95% CI: − 7.332 to − 1.648, *P* = 0.002) and brachial DBP (− 3.629 mmHg, 95% CI: − 5.825 to − 1.434, *P* = 0.001). Nine of the trials were meta-analyzed for aortic BP which showed that L-Cit intervention significantly reduced aortic SBP (− 6.763 mmHg, 95% CI: − 10.991 to − 2.534, *P* = 0.002), but not aortic DBP (− 3.396 mmHg, 95% CI: − 7.418 to 0.627, *P* = 0.098). The observed reducing effects of L-Cit appeared stronger for non-resting than for resting brachial SBP (*P* for difference = 0.044).

**Conclusion:**

L-Cit supplementation significantly decreased non-resting brachial and aortic SBP. Brachial DBP was significantly lowered by L-Cit regardless of resting status. Given the relatively small number of available trials in the stratified analyses and the potential limitations of these trials, the present findings should be interpreted cautiously and need to be confirmed in future well-designed trials with a larger sample size.

## Background

Globally, approximately 20–50% of adults are affected by hypertension, a major powerful predictor of cardiovascular disease (CVD) and premature death [1]. Reduction of blood pressure (BP) is clinically crucial for reducing risk of CVD, and even a modest reduction can still confer cardiovascular benefits [2]. However, it is estimated that nearly 50% of those treated for hypertension have uncontrolled resting BP [3].

Generally, brachial BP is used for the diagnosis and management of hypertension. However, brachial BP does not reflect aortic BP and cardiac load accurately [4]. It has been demonstrated that aortic BP and brachial BP have substantially different responses to BP-lowering drugs [5]. Furthermore, BP post isometric handgrip (IGH) [6], whole-body vibration training (WBVT) [7] and cold exposure [8] can be increased via sympathetic and muscle metaboreflex-mediated vasoconstriction compared with baseline BP. Adults with hypertension always have exaggerated exercise BP due to muscle metaboreflex overactivation [6]. This augmented exercise BP, which is not controlled by BP-lowering drugs, is an independent risk factor for cardiovascular events and mortality [9].

L-citrulline (L-Cit), a colorless, water-soluble amino acid, is an effective endogenous precursor of L-Arginine (L-Arg) [10] and nitric oxide (NO) [11, 12]. Oral L-Arg has been shown to lower BP [13]. Thus, it is biologically plausible that L-Cit may also have BP-lowering potential. A number of randomized controlled trials (RCTs) have evaluated the effects of L-Cit on BP in subjects with varying background characteristics, but the results are inconsistent [11, 12, 14–16].

Three meta-analyses [17–19] have investigated the effect of L-Cit supplementation on BP. Two [17, 18] were conducted to evaluate the effect of L-Cit on brachial BP. The present study added the analysis on aortic BP, since aortic BP is more relevant for cardiovascular risk and responsive to antihypertensive treatments than brachial BP [4, 5]. Although another review [19] took both resting brachial and aortic BP into account, an update is needed because eligible trials were missed [15, 16, 20]. Previous meta-analyses have not evaluated the influence of L-Cit on BP during conditions with increased sympathetic-mediated vasoconstriction such as exercise and cold exposure.

We performed this systematic review with meta-analysis to evaluate the effect of L-Cit supplementation on brachial and aortic BP, taking the differences between resting BP and non-resting BP into account.

## Methods

### Literature search

This systematic review was performed in accordance with the principles outlined in the Cochrane Handbook 5.1.0 [21] for systematic reviews of interventions. We searched multiple electronic databases including PubMed, Embase, Cochrane Library, Web of Science, and Clinical Trials for potentially relevant studies. Articles published from inception to May 5, 2019 were filtered, and search strategies are reported in Additional file 17: Table S1. In addition, reference lists of relevant trials were searched to identify more potentially eligible trials. Our search was restricted to studies published in English. Two researchers (H-HY and X-LL) independently performed the search and justification for eligibility, and any disagreement was resolved by consensus.

### Inclusion and exclusion criteria

Studies were included if they met all of the following criteria: 1) the design was a RCT; 2) the participants were adults aged 18 years or older; 3) L-Cit was given as the only intervention by oral administration; 4) the intervention lasted at least for one week; and 5) sufficient data on BP at baseline and at the end of follow-up or the changes of BP in each group were reported. Conference abstracts or studies lacking a controlled group were not considered.

### Data extraction

The following data were extracted from each of the included trials: the first author’s name, year of publication, study location, study design, blinding status, sample size, dose of L-Cit, intervention duration, participants’ characteristics including sex, age, health status and BP values.

### Quality evaluation

According to RevMan (Cochrane Review Manager, version 5.3), several trial components including “random sequence generation”, “allocation concealment”, “blinding of participants and personnel”, “blinding of outcome assessment”, “incomplete outcome data”, “selective reporting” and “other bias” were used to assess the quality of the included RCTs. The included trials were categorized as having ‘high’, ‘low’, or ‘unclear’ risk of bias according to the assessment criteria.

### Data synthesis

STATA 14.0 (Stata Corp., College Station, TX) was used for data analyses. For parallel trials, the net changes of each outcome in intervention and control groups were calculated as differences between mean values before and after treatments. For crossover trials, the net changes were calculated as the difference in the post-treatment values of each group. Standard deviations (SDs) of BP net changes, if not reported, were calculated by the following formulas according to the Cochrane Handbook 16.1.3.2 [21]:

$$ \boldsymbol{R}=\frac{{{\boldsymbol{SD}}_{\boldsymbol{Experimen}}}^{\mathbf{2}}+{{\boldsymbol{SD}}_{\boldsymbol{Control}}}^{\mathbf{2}}-{{\boldsymbol{SD}}_{\boldsymbol{Change}}}^{\mathbf{2}}}{\mathbf{2}{\boldsymbol{SD}}_{\boldsymbol{Experimen}}{\boldsymbol{SD}}_{\boldsymbol{Control}}} $$

$$ \boldsymbol{SD}=\sqrt{{{\boldsymbol{SD}}_{\boldsymbol{Experimen}}}^{\mathbf{2}}+{{\boldsymbol{SD}}_{\boldsymbol{Co}n\boldsymbol{trol}}}^{\mathbf{2}}-\left(\mathbf{2}\boldsymbol{R}{\boldsymbol{SD}}_{\boldsymbol{Experimen}}{\boldsymbol{SD}}_{\boldsymbol{Co}\boldsymbol{ntrol}}\right)} $$

Heterogeneity was quantified by the Cochrane *Q* in addition to the *I*^*2*^ statistics. A fixed-effect meta-analysis was performed to pool the weighted mean difference (WMD) between experiment and control groups because of limited heterogeneity observed across the analyses. Stratified analyses by sex, age and BP resting status were also conducted, followed by meta-regression analyses exploring potential sources of heterogeneity. Both Begg’s rank correlation and Egger’s linear regression tests were conducted to assess the potential publication bias. *P* < 0.05 was considered statistically significant.

## Results

### Study selection

**Fig. 1:**
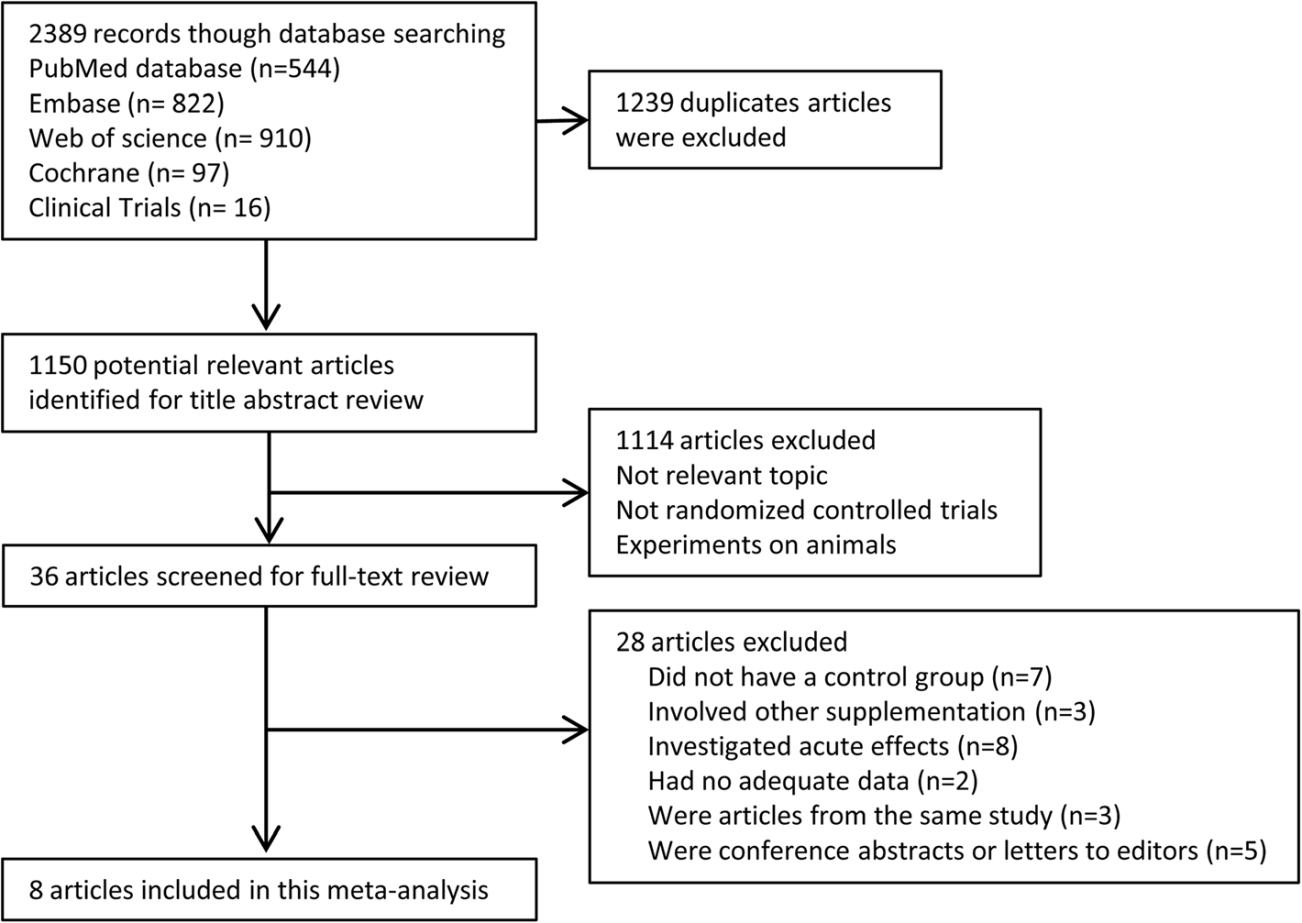
Flow chart of study screening and selection

The initial search yielded a total of 2389 records, of which 1150 independent records were screened after removing the duplicates. By reading further the abstracts, 36 relevant articles remained for full-text evaluations. Among these, 28 studies were further excluded because they did not have a control group (*n* = 7), used phytochemicals combined with L-Arg or watermelon (*n* = 3), investigated acute effects (intervention duration < 1 week) (*n* = 8), had no adequate data (*n* = 2), were articles from the same study (n = 3), were conference abstracts or letters to editors (*n* = 5). Finally, eight eligible studies [11, 12, 14–16, 20, 22, 23], including 14 independent trials, were included in the present meta-analysis. Study selection process is described in Fig. 1.

### Study characteristics

Various characteristics of the included studies were summarized in Table 1. These studies were published between 2010 and 2017. Six studies [12, 14, 16, 20, 22, 23] were conducted in the US, one [15] in Mexico and one [11] in Japan. Five of the six US studies were from Florida, and we confirmed that they were independent of each other based on the article review in addition to correspondences with relevant authors. Four studies [11, 12, 15, 20] employed parallel design, others were crossover designed studies with a 2-week washout period. Mean age of the participants ranged from 22 to 70 years. Healthy [11, 14, 16, 22], hypertensive [12, 20, 23] or systolic heart failure [15] participants were recruited in these studies. Capsules provided by Kyowa Hakko Bio Co., Ltd. (Tokyo, Japan) or NOW Foods (Bloomingdale, IL) were supplemented in most studies, and L-Cit drinks were taken in one study [15]. Dosage of L-Cit ranged from 3 g/day to 11 g/day. Intervention durations varied considerably from one week to 16 weeks.

**Table 1:** Baseline characteristics of the studies included in the meta-analysis

Author/Year	Countries	Design	Size (M/F)	Age (year±SD)	Status	Dose (g/day)	Duration (wks)	Baseline BP (mm Hg)	
								L- Cit	Placebo
Gonzales 2017	USA	X, DB	12/13	70 ± 5	Healthy/ Hypertension	6	2	M: 130 ± 13/65 ± 8 F: 137 ± 16/77 ± 9	
Figueroa 2016	USA	X, DB	16/0	24 ± 8	Healthy	6	2	123 ± 12/68 ± 8	122 ± 8/67 ± 4
Wong 2016	USA	P	0/23	58 ± 4.8	Healthy/ Hypertension	6	8	138 ± 4/81 ± 4	137 ± 4/80 ± 3
Wong 2015	USA	P	0/27	58 ± 3	Healthy/ Hypertension	6	8	140 ± 9/78 ± 7	141 ± 2/80 ± 8
Sanchez-Gonzalez 2013	USA	X	16/0	23 ± 12	Healthy	7–11	2	116 ± 2/59 ± 3	
Balderas-Munoz 2012	Mexico	P, DB	24/11	67 ± 9	Systolic heart failure	3	16	113 ± 17/70 ± 12	118 ± 16/77 ± 11
Ochiai 2012	Japan	P, DB	15/0	58.3 ± 4.4	Healthy	5.6	1	136 ± 13/82 ± 7	131 ± 9/85 ± 7
Figueroa 2010	USA	X, DB	17/0	22 ± 4.1	Healthy	6	4	120 ± 12/67 ± 8	121 ± 12/68 ± 8

*BP* blood pressure; *DB* double blinding; *F* female; *L- Cit* L-citrulline; *M* male; *P* parallel controlled trial; *SD* standard deviation; *X* cross-over study design

**Fig. 2:**
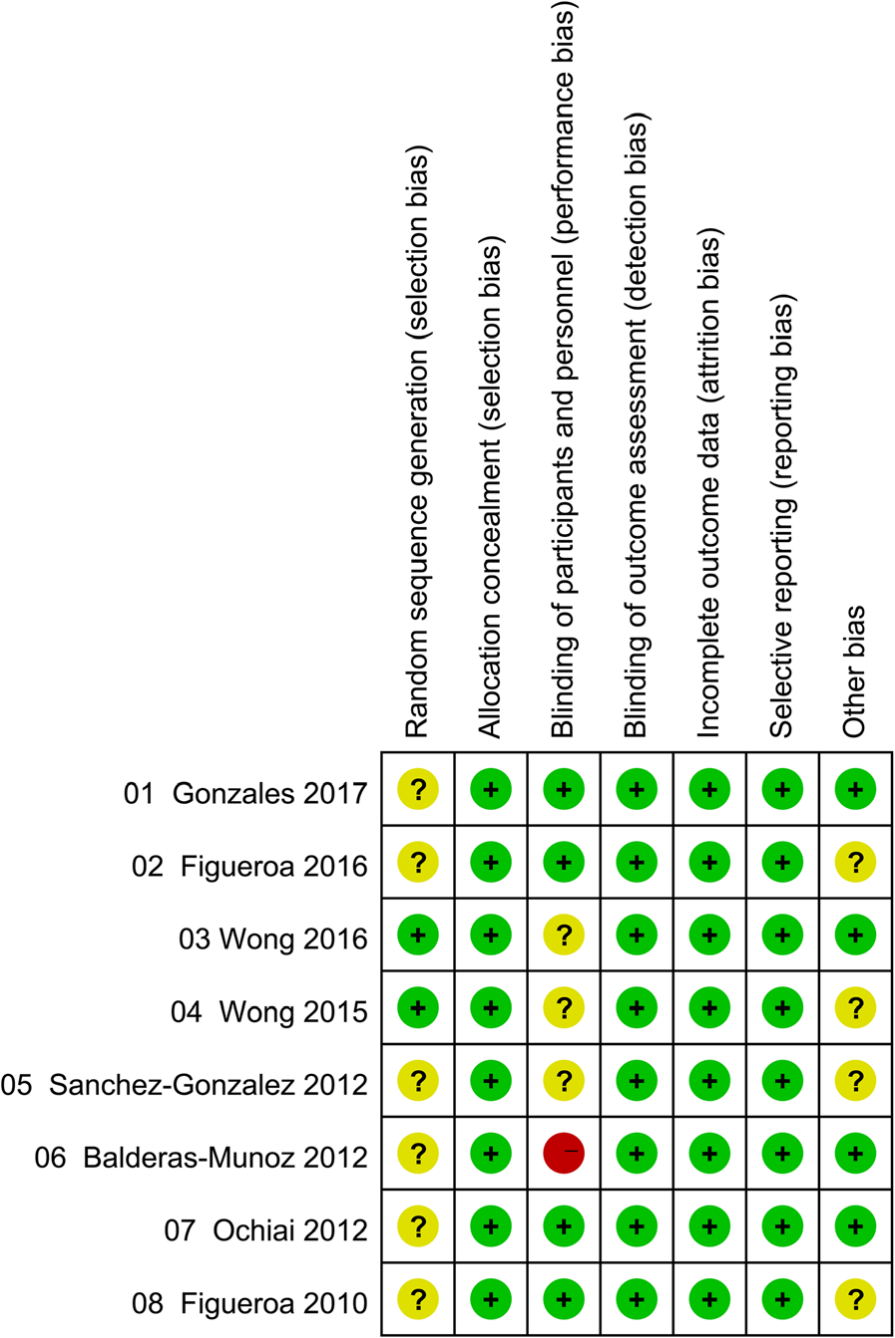
The summary of review authors’ judgments about each risk of bias item for each included study. Note: “+”: low risk; “?”: unclear risk; “-”: high risk

Results for risk-of-bias assessment of the included studies are summarized in Fig. 2. Although “randomization” was described, description of randomization method was lacking among six studies [11, 14–16, 22, 23] . The study by Balderas-Munoz et al. [15] had a high risk of bias on “blinding of participants and personnel” due to lack of placebo supplementation for the control group. And three studies [12, 16, 20] had unclear biases on this term since they did not clarify the blinding method. There might potentially be “other bias” [12, 14, 16, 22], because an investigation of resting and non-resting BP was conducted simultaneously. Fortunately, there were no risk of bias on “allocation concealment”, “blinding of outcome assessment”, “incomplete outcome data”, or “selective reporting”. In addition, there was a suggestion of publication bias regarding the effect of L-Cit on brachial DBP by the Begg’s test (*P* = 0.025), and on aortic DBP by both Begg’s and Egger’s tests (Begg, *P* = 0.029; Egger, *P* = 0.015). The overall estimates remained unchanged after using the “trim-and-fill” method to adjust for the bias.

### Meta-analysis of L-Cit supplementation on BP

Compared to control, oral L-Cit resulted in BP changes from − 14.00 to 4.00 mmHg, from − 16.00 to 2.10 mmHg, from − 15.00 to 3.00 mmHg, and from − 15.00 to 2.00 mmHg in brachial SBP, brachial DBP, aortic SBP, and aortic DBP, respectively. Results of meta-analyses showed that L-Cit supplement significantly reduced brachial SBP, brachial DBP, aortic SBP, but not aortic DBP. The estimated overall WMDs were: − 4.49 mmHg (95% CI: − 7.33 to − 1.65) for brachial SBP (Fig. 3-A), − 3.63 mmHg (95% CI: − 5.82 to − 1.43) for brachial DBP (Fig. 3-B), − 6.76 mmHg (95% CI: − 10.99 to − 2.53) for aortic SBP (Fig. 3-C), and − 3.40 mmHg (95% CI: − 7.42 to 0.63) for aortic DBP (Fig. 3-D).

**Fig. 3-A:**
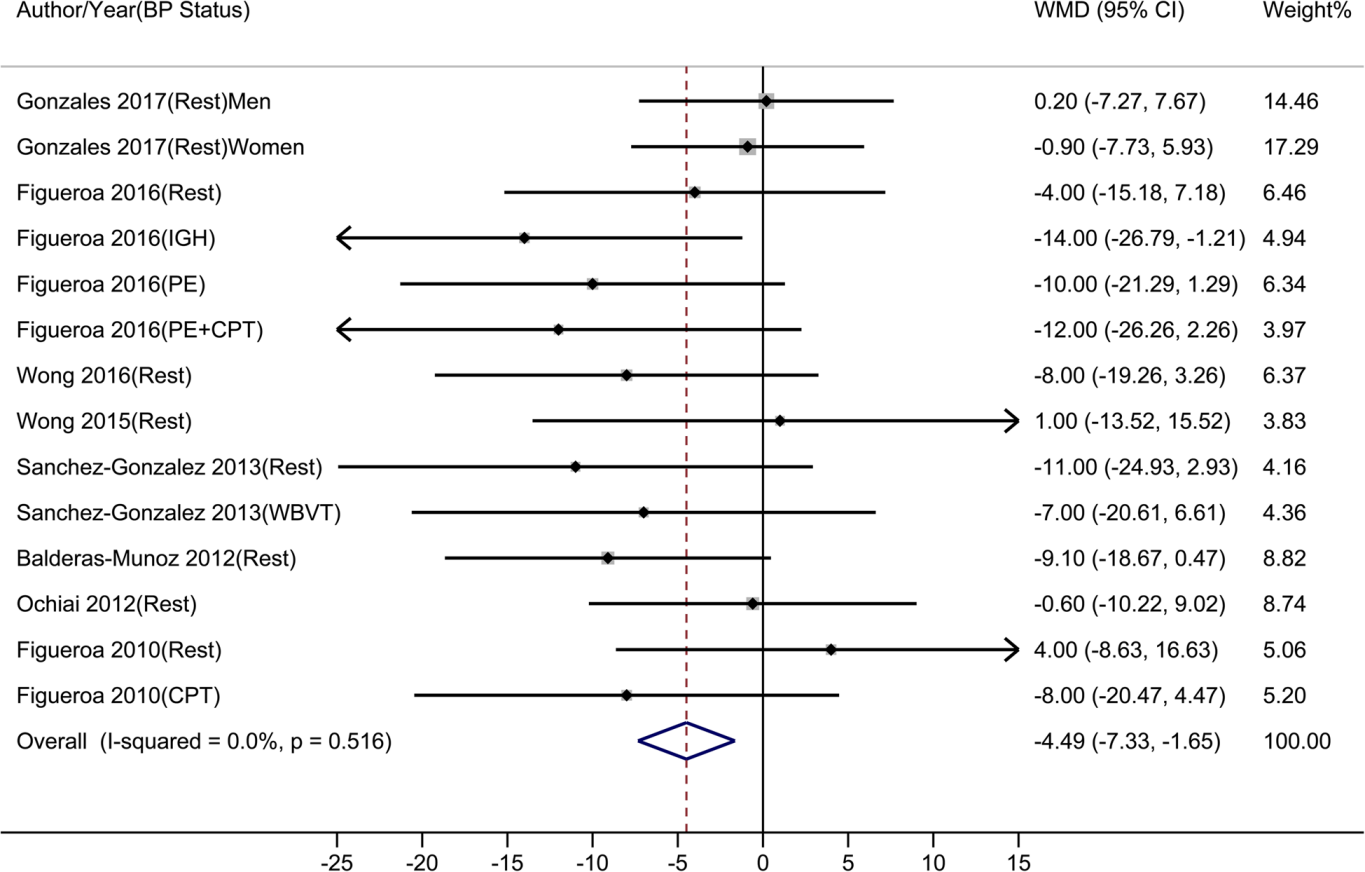
Meta-analysis of the effect of L-Citrulline on brachial systolic blood pressure Abbreviations: CPT, cold pressure test; IHG, isometric handgrip; PE, post-exercise muscle ischemia (metaboreflex); WBVT, whole-body vibration training; WMD, weighted mean difference

**Fig. 3-B:**
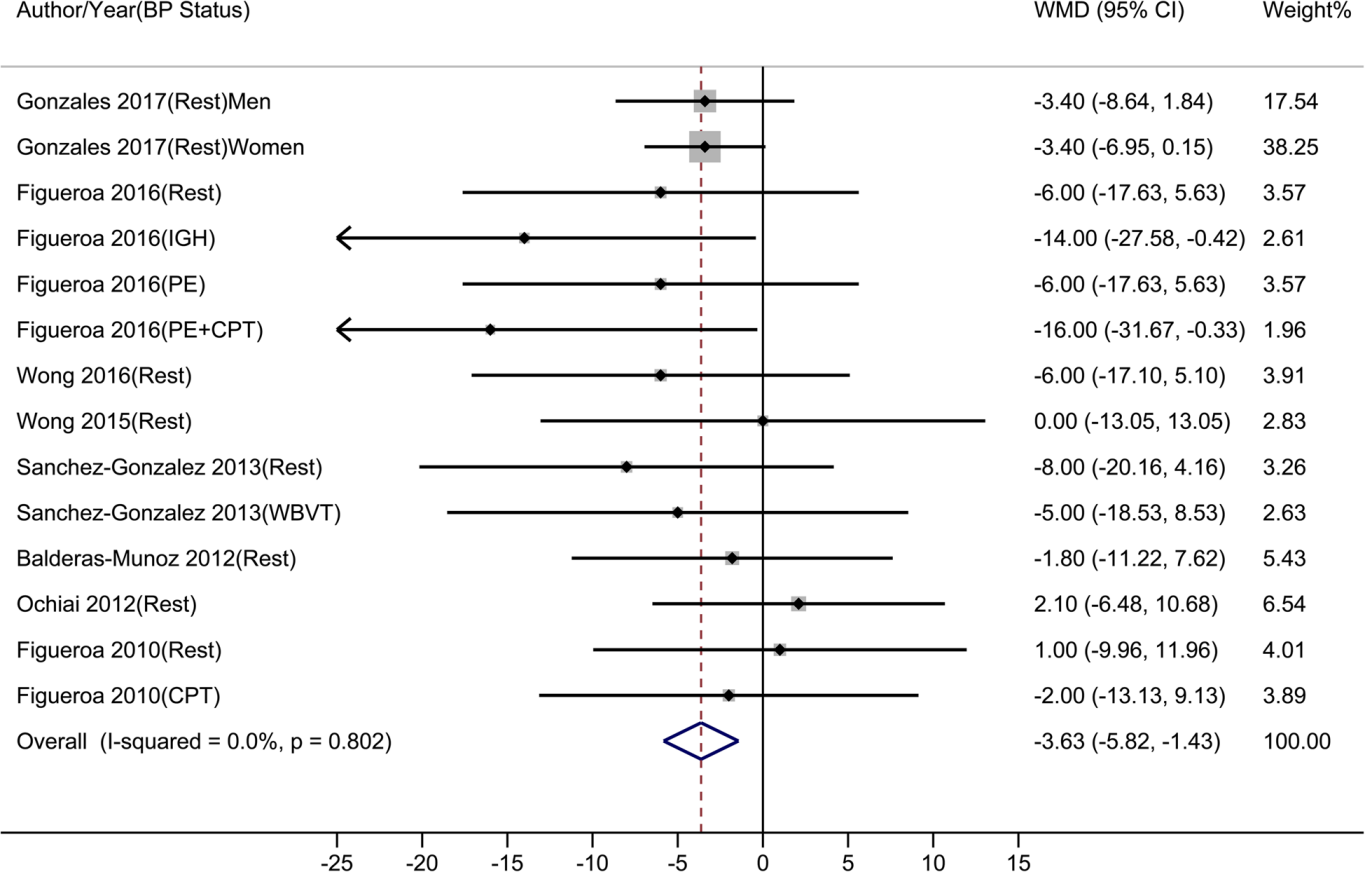
Meta-analysis of the effect of L-Citrulline on brachial diastolic blood pressure Abbreviations: CPT, cold pressure test; IHG, isometric handgrip; PE, post-exercise muscle ischemia (metaboreflex); WBVT, whole-body vibration training; WMD, weighted mean difference

**Fig. 3-C:**
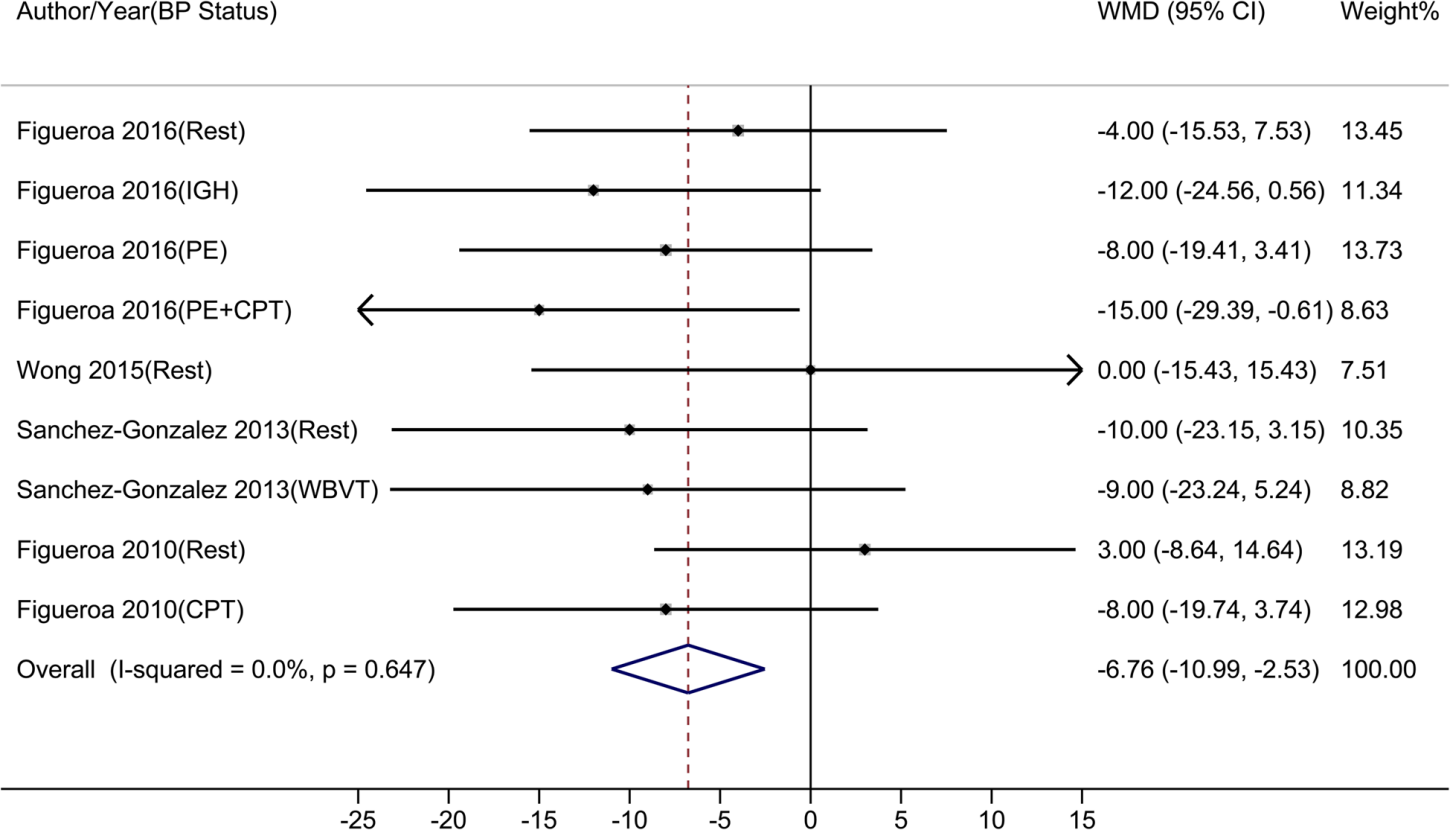
Meta-analysis of the effect of L-Citrulline on aortic systolic blood pressure Abbreviations: CPT, cold pressure test; IHG, isometric handgrip; PE, post-exercise muscle ischemia (metaboreflex); WBVT, whole-body vibration training; WMD, weighted mean difference

**Fig. 3-D:**
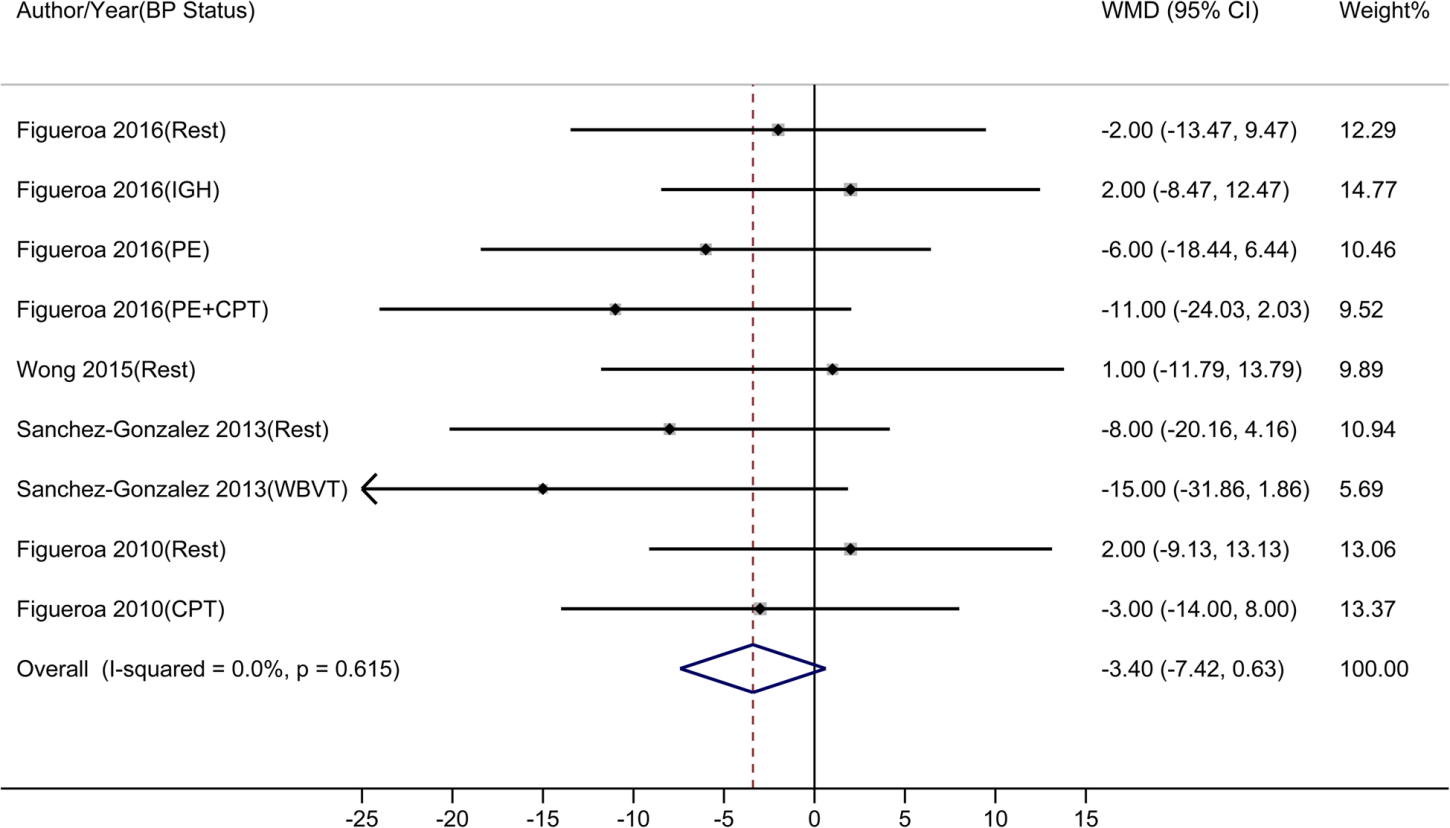
Meta-analysis of the effect of L-Citrulline on aortic diastolic blood pressure Abbreviations: CPT, cold pressure test; IHG, isometric handgrip; PE, post-exercise muscle ischemia (metaboreflex); WBVT, whole-body vibration training; WMD, weighted mean difference

### Stratified and meta-regression analyses

**Fig. 4:**
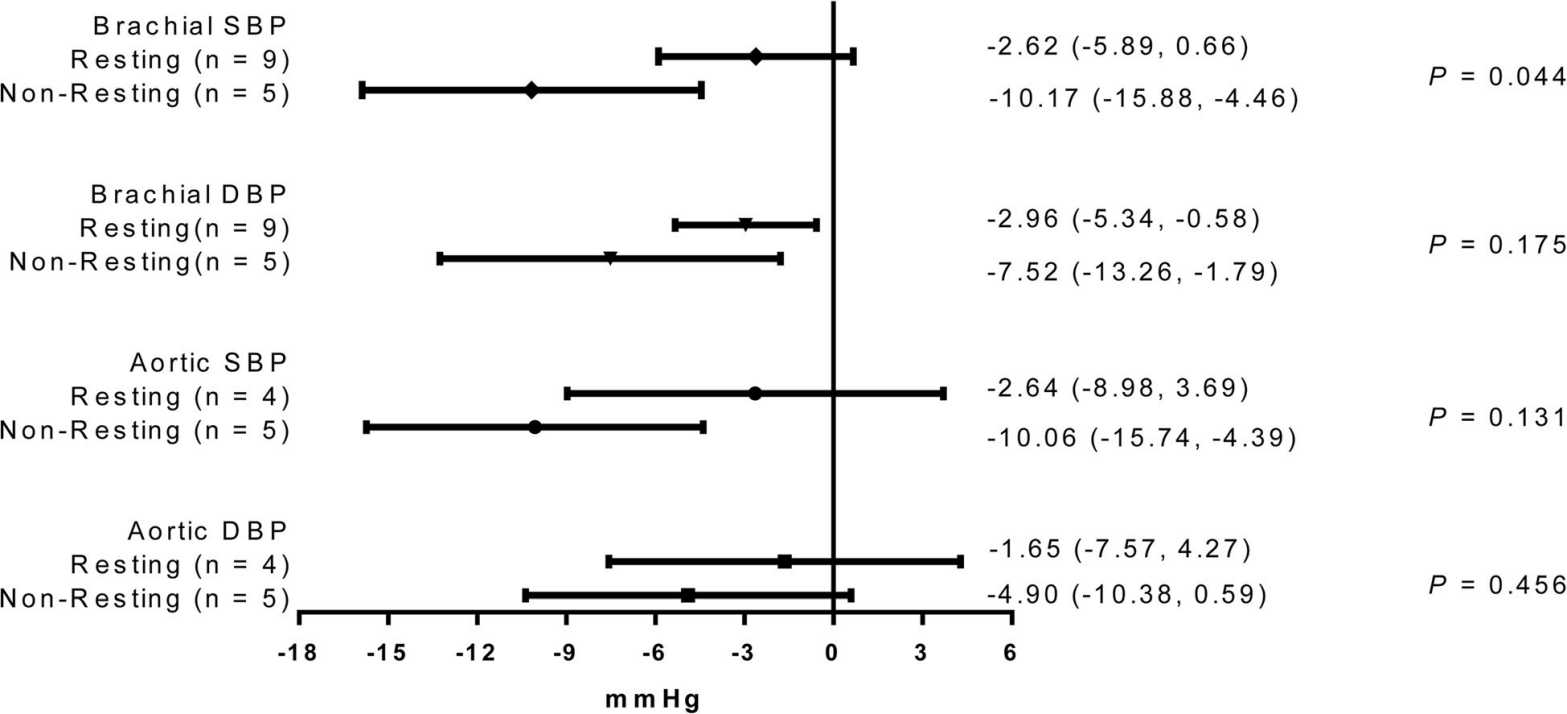
Analyses of L-citrulline supplementation on brachial and aortic BP according to BP status. Abbreviations: BP, blood pressure; DBP, diastolic blood pressure; P, test for difference between subgroups; SBP, systolic blood pressure

As shown in Fig. 4, the estimated reduction of non-resting brachial SBP was − 10.17 mmHg (95% CI: − 15.88 to − 4.46), with no significant reduction of resting brachial SBP (− 2.62 mmHg, 95% CI: − 5.89, 0.66). Meta-regression analyses showed that the reducing effects of L-Cit was stronger for non-resting SBP than for resting brachial SBP (*P* for difference = 0.044) (Additional file 1: Figure S1 and Additional file 2: Figure S2). Both non-resting brachial DBP (− 7.52 mmHg, − 13.26 to − 1.79) and resting brachial DBP (− 2.96 mmHg, − 5.34 to − 0.58) were significantly lowered by L-Cit supplementation (*P* for difference = 0.175) (Additional file 3**:** Figure S3 and Additional file 4**:** Figure S4). Similar to the effect on brachial SBP, L-Cit supplementation significantly decreased non-resting aortic SBP by 10.06 mmHg (95% CI: − 15.74 to − 4.39), and had no significant effect on resting aortic SBP (− 2.64 mmHg, 95% CI: − 8.98, 3.69). However, no significant difference was observed between them (*P* for difference = 0.131) (Additional file 5: Figure S5 and Additional file 6: Figure S6). L-Cit supplementation had no significant effect on non-resting aortic DBP (− 4.90 mmHg, − 10.38 to 0.59) or resting aortic DBP (− 1.65 mmHg, − 7.57 to 4.27) (*P* for difference = 0.456) (Additional file 7: Figure S7 and Additional file 8: Figure S8).

Stratified analyses found that brachial SBP was significantly reduced among men and those aged < 30 years, but not among women or participants > 50 years. Consistent with an overall estimated WMD, brachial DBP was significantly lowered in both men and women, as well as both age groups. No heterogeneity between groups was found in the above analysis (*P* > 0.05) (Table 2, Additional file 9: Figure S9, Additional file 10: Figure S10, Additional file 11: Figure S11, Additional file 12: Figure S12). We did not perform stratified analyses for aortic BP due to small number of studies.

**Table 2:** Sex- and age-stratified analysis of the effect of L-Citrulline on brachial systolic and diastolic blood pressure

	Brachial SBP		Brachial DBP	
	WMD (95% CI)	*P*	WMD (95% CI)	*P*
Gender				
Men	−4.81 (−8.37, −1.24	0.246	−4.03 (−7.14, −0.91)	0.998
Women	−2.28 (− 7.70, 3.14)		−3.41 (−6.69, − 0.14)	
Age (Years old)				
> 50	−2.44 (− 6.13, 1.24)	0.113	− 2.81 (−5.35, − 0.26)	0.234
< 30	−7.50 (− 11.97, − 3.03)		−6.03 (− 10.38, − 1.68)	

*BP* blood pressure; *P* difference between groups by meta-regression analyses; *WMD* weighted mean difference

In addition, a sensitivity analysis in which studies were excluded one by one was conducted. The summary WMD ranged from − 5.28 (95% CI: − 8.36, − 2.21) to − 4.00 (95% CI: − 6.91, − 1.08) for brachial SBP, − 4.03 (95% CI: − 6.30, − 1.76) to − 3.35 (95% CI: − 5.58, − 1.13) for brachial DBP, − 8.25 (95% CI: − 12.78, − 3.71) to − 5.98 (95% CI: − 10.41, − 1.56) for aortic SBP, and − 4.33 (95% CI: − 8.69, 0.03) to − 2.60 (95% CI: − 6.82, 1.63) for aortic DBP. In summary, the overall estimated WMD of neither brachial BP nor aortic BP were remarkably altered by excluding any single trial (Additional file 13: Figure S13, Additional file 14: Figure S14, Additional file 15: Figure S15, Additional file 16: Figure S16).

## Discussion

Findings from this meta-analysis of 14 trials indicated that L-Cit supplementation had significant effects on lowering brachial BP and aortic SBP, but no effect on aortic DBP. Further analysis according to resting status revealed that L-Cit reduced non-resting brachial and aortic SBP, as well as resting and non-resting brachial DBP, but no effect was found on aortic DBP. Subgroup analyses showed that L-Cit supplementation reduced brachial SBP in men and those aged < 30 years, and reduced brachial DBP among both genders and age groups.

### Results from previous meta-analyses

There are some differences between our analysis and the previous three meta-analyses. It is notable that in addition to L-Cit, watermelon also contains other bioactive compounds, such as L-Arg and lycopene. Therefore, we treated L-Cit rather than watermelon as the only supplementation, which differed from the meta-analysis of Mahboobi et al. [17]. Furthermore, Mirenayat et al. [19] analyzed resting BP in five trials and found that L-Cit supplementation had no beneficial effect on either brachial BP or aortic BP. In the present study, we added three more eligible studies [15, 16, 20], and analyzed the effects of L-Cit supplementation on brachial BP and aortic BP according to resting status. Furthermore, meta-regression analyses were carried out to explore potential sources of heterogeneity. Thus, we provided more informative evidence for the effects of L-Cit on BP in this updated meta-analysis.

### Results from studies that were not eligible for this meta-analysis

Some studies were excluded from the present meta-analysis because of unsuitable supplementary methods [24, 25], no control group [10, 26], being an acute L-Cit administration [27] or review [28]. Most of these studies illustrated the beneficial effects of L-Cit intervention on BP, except for one [24]**,** which showed no significant effect on resting brachial BP in normotensives. Churchward-Venne et al. [25] reported that a single dose of 10 g L-Cit supplementation co-ingested with whey protein may attenuate BP responses to exercise but did not reduce resting BP in healthy elderly men. Besides exercise BP, Morita et al. [10] found that oral administration of 800 mg of L-Cit for eight weeks resulted in a moderate reduction of resting DBP in adults with vasospastic angina. Orozco-Gutierrez et al. [26] also demonstrated that resting brachial BP was significantly lowered after two months of L-Cit administration. Alsop et al. [27] showed that 3 g/d of L-Cit supplementation for one week significantly decreased brachial SBP by 6% and brachial DBP by 14% [28]. These preclinical trials supported the hypothesis that L-Cit supplementation may possess the capability to reduce BP.

### Findings from animal studies

Results from animal studies on the effects of L-Cit supplementation on BP control are conflicting. An impaired citrulline-arginine production pathway in the kidneys decreases renal NO, leading to the development of hypertension in spontaneously hypertensive rats (SHRs) [29]. Koeners et al. [29] showed that 2.5 g/L of L-Cit supplementation from day seven of gestation to six weeks old of the offspring reduced SBP of both female and male SHRs. Chien et al. [30] showed that 0.25% L-Cit supplementation for eight weeks prevented the transition from prehypertension to hypertension in young hypertensive rats. However, in the study by Mor et al. [31], intravenously administered L-Cit (10–300 μg/kg/min) did not affect SBP in male rats. Interestingly, Tain et al. found that a 0.25% L-Cit solution supplemented during the whole period of pregnancy and lactation prevented NG-nitro-L-arginine-methyl ester (NO synthase inhibitor)-induced hypertension in the young offspring rats [32]; but, the intervention raised their BP when they reached adulthood [33].

### Potential mechanisms

L-Cit is an effective exogenous precursor of L-Arg, a natural substrate for NO [34]. As an endothelium-derived relaxing factor, NO can induce vascular smooth muscle relaxation and vasodilation [35]. Consequently, endothelial-mediated vasodilation leads to BP reduction. In our previous meta-analysis including 11 RCTs, we found that oral L-Arg significantly lowered brachial SBP by 5.39 mmHg and brachial DBP by 2.66 mmHg [13]. The bioavailability of oral L-Cit may be higher than that of oral L-Arg, given the fact that L-Cit bypasses splanchnic extraction and catabolism by the enzyme arginase located in the enterocytes of intestines, liver, and vasculature [36, 37].

In this regard, several mechanisms underlying the BP-lowering effect of L-Cit supplementation have been proposed, including: 1) up-regulation of endothelial-NO synthase (eNOS) [10], a key enzyme for NO production; 2) effective absorption and conversion to L-Arg [38], which is catabolized by eNOS to NO in endothelial cells [11, 12, 39]; 3) inhibition of arginase, a catabolic enzyme that reduces L-Arg bioavailability by converting L-Arg to ornithine and urea [38, 39]; and 4) reduction of asymmetric dimethyl arginine [10], an eNOS competitive inhibitor, to enhance NO production [11, 12, 30, 37].

In addition, a stronger effect of oral L-Cit was observed on non-resting BP, including during the post-exercise period, metaboreflex activation, and cold exposure. Elevated BP response to exercise is probably caused by inadequate perfusion of the active muscles, which is a consequence of a mismatch between supply and demand mediated by increased sympathetic vasoconstriction [9]. In this condition, L-Cit supplementation may attenuate exercise BP responses by improvements in regulatory mechanisms of the vascular tone including sympathetic activity and endothelial NO production [10, 12, 20]. On the other hand, BP response to cold exposure was related to reduced cold-induced systemic vascular reactivity [16, 22].

### Limitations

Some limitations of our meta-analysis need to be discussed. First, additional stratified-analysis according to other participant or trial characteristics such as race/ethnicity, health status, dose and duration of intervention were not performed due to the limited number of eligible trials. Dose-response relationship between BP reduction and baseline BP is also yet to be elucidated. Second, the reliability of the results was lowered by the low-quality trials that were included. Nevertheless, sensitivity analyses did not suggest heterogeneity caused by those low-quality trials. Third, potential impacts of publication bias on our findings cannot be excluded, given the tendency to publish larger studies or small studies with encouraging findings.

## Conclusion

L-Cit supplementation significantly decreased non-resting brachial and aortic SBP. Brachial DBP was significantly lowered by L-Cit regardless of resting status. Given the relatively small number of available trials in the stratified analyses and the potential limitations of these trials, the present findings should be interpreted cautiously and need to be confirmed in future well-designed trials with a larger sample size.

## Supplementary information

**Additional file 1: Figure S1.** Stratified analysis of the effect of L-Citrulline on brachial systolic blood pressure according to BP status. Abbreviations: CPT, cold pressure test; IHG, isometric handgrip; PE, post-exercise muscle ischemia (metaboreflex); WBVT, whole-body vibration training; WMD, weighted mean difference.

**Additional file 2: Figure S2.** Meta-regression analysis of brachial systolic blood pressure according to BP status. Abbreviations: CPT, cold pressure test; IHG, isometric handgrip; PE, post-exercise muscle ischemia (metaboreflex); WBVT, whole-body vibration training; WMD, weighted mean difference.

**Additional file 3: Figure S3.** Stratified analysis of the effect of L-Citrulline on brachial diastolic blood pressure according to BP status. Abbreviations: CPT, cold pressure test; IHG, isometric handgrip; PE, post-exercise muscle ischemia (metaboreflex); WBVT, whole-body vibration training; WMD, weighted mean difference.

**Additional file 4: Figure S4.** Meta-regression analysis of brachial diastolic blood pressure according to BP status. Abbreviations: CPT, cold pressure test; IHG, isometric handgrip; PE, post-exercise muscle ischemia (metaboreflex); WBVT, whole-body vibration training; WMD, weighted mean difference.

**Additional file 5: Figure S5.** Stratified analysis of the effect of L-Citrulline on aortic systolic blood pressure according to BP status. Abbreviations: CPT, cold pressure test; IHG, isometric handgrip; PE, post-exercise muscle ischemia (metaboreflex); WBVT, whole-body vibration training; WMD, weighted mean difference.

**Additional file 6: Figure S6.** Meta-regression analysis of aortic systolic blood pressure according to BP status. Abbreviations: CPT, cold pressure test; IHG, isometric handgrip; PE, post-exercise muscle ischemia (metaboreflex); WBVT, whole-body vibration training; WMD, weighted mean difference.

**Additional file 7: Figure S7.** Stratified analysis of the effect of L-Citrulline on aortic diastolic blood pressure according to BP status. Abbreviations: CPT, cold pressure test; IHG, isometric handgrip; PE, post-exercise muscle ischemia (metaboreflex); WBVT, whole-body vibration training; WMD, weighted mean difference.

**Additional file 8: Figure S8.** Meta-regression analysis of aortic diastolic blood pressure according to BP status. Abbreviations: CPT, cold pressure test; IHG, isometric handgrip; PE, post-exercise muscle ischemia (metaboreflex); WBVT, whole-body vibration training; WMD, weighted mean difference.

**Additional file 9: Figure S9.** Sex-stratified analysis of the effect of L-Citrulline on brachial systolic blood pressure. Abbreviations: CPT, cold pressure test; IHG, isometric handgrip; PE, post-exercise muscle ischemia (metaboreflex); WBVT, whole-body vibration training; WMD, weighted mean difference.

**Additional file 10: Figure S10.** Age-stratified analysis of the effect of L-Citrulline on brachial systolic blood pressure. Abbreviations: CPT, cold pressure test; IHG, isometric handgrip; PE, post-exercise muscle ischemia (metaboreflex); WBVT, whole-body vibration training; WMD, weighted mean difference.

**Additional file 11: Figure S11.** Sex-stratified analysis of the effect of L-Citrulline on brachial diastolic blood pressure. Abbreviations: CPT, cold pressure test; IHG, isometric handgrip; PE, post-exercise muscle ischemia (metaboreflex); WBVT, whole-body vibration training; WMD, weighted mean difference.

**Additional file 12: Figure S12.** Age-stratified analysis of the effect of L-Citrulline on brachial diastolic blood pressure. Abbreviations: CPT, cold pressure test; IHG, isometric handgrip; PE, post-exercise muscle ischemia (metaboreflex); WBVT, whole-body vibration training; WMD, weighted mean difference.

**Additional file 13: Figure S13.** Sensitivity analysis of the effect of L-Citrulline on brachial systolic blood pressure. Abbreviations: CPT, cold pressure test; IHG, isometric handgrip; PE, post-exercise muscle ischemia (metaboreflex); WBVT, whole-body vibration training.

**Additional file 14: Figure S14.** Sensitivity analysis of the effect of L-Citrulline on brachial diastolic blood pressure. Abbreviations: CPT, cold pressure test; IHG, isometric handgrip; PE, post-exercise muscle ischemia (metaboreflex); WBVT, whole-body vibration training.

**Additional file 15: Figure S15.** Sensitivity analysis of the effect of L-Citrulline on aortic systolic blood pressure. Abbreviations: CPT, cold pressure test; IHG, isometric handgrip; PE, post-exercise muscle ischemia (metaboreflex); WBVT, whole-body vibration training.

**Additional file 16: Figure S16.** Sensitivity analysis of the effect of L-Citrulline on aortic diastolic blood pressure. Abbreviations: CPT, cold pressure test; IHG, isometric handgrip; PE, post-exercise muscle ischemia (metaboreflex); WBVT, whole-body vibration training.

**Additional file 17: Table S1**. Summarised search strategies to identify the effects of L-citrulline interventions on blood pressure. (DOCX 15 kb)

## Data Availability

The datasets used and/or analyzed during the current study are available from the manuscript and the corresponding author on reasonable request.

## Abbreviations

BP: Blood pressure
CVD: Cardiovascular disease
DBP: Diastolic blood pressure
eNOS: Endothelial-NO synthase
IGH: Isometric handgrip
L-Arg: L-Arginine
L-Cit: L-citrulline
NO: Nitric oxide
RCTs: Randomized controlled trials
SBP: Systolic blood pressure
SDs: Standard deviations
SHRs: Spontaneously hypertensive rats
WBVT: Whole-body vibration training
WMD: Weighted mean difference
