# Supplementary figures and images for: Effect of oral L-citrulline on brachial and aortic blood pressure defined by resting status: evidence from randomized controlled trials
Source: Nutr Metab (Lond). 2019 Dec 26;16:89. doi: 10.1186/s12986-019-0415-y (PMC6933755; doi:10.1186/s12986-019-0415-y)

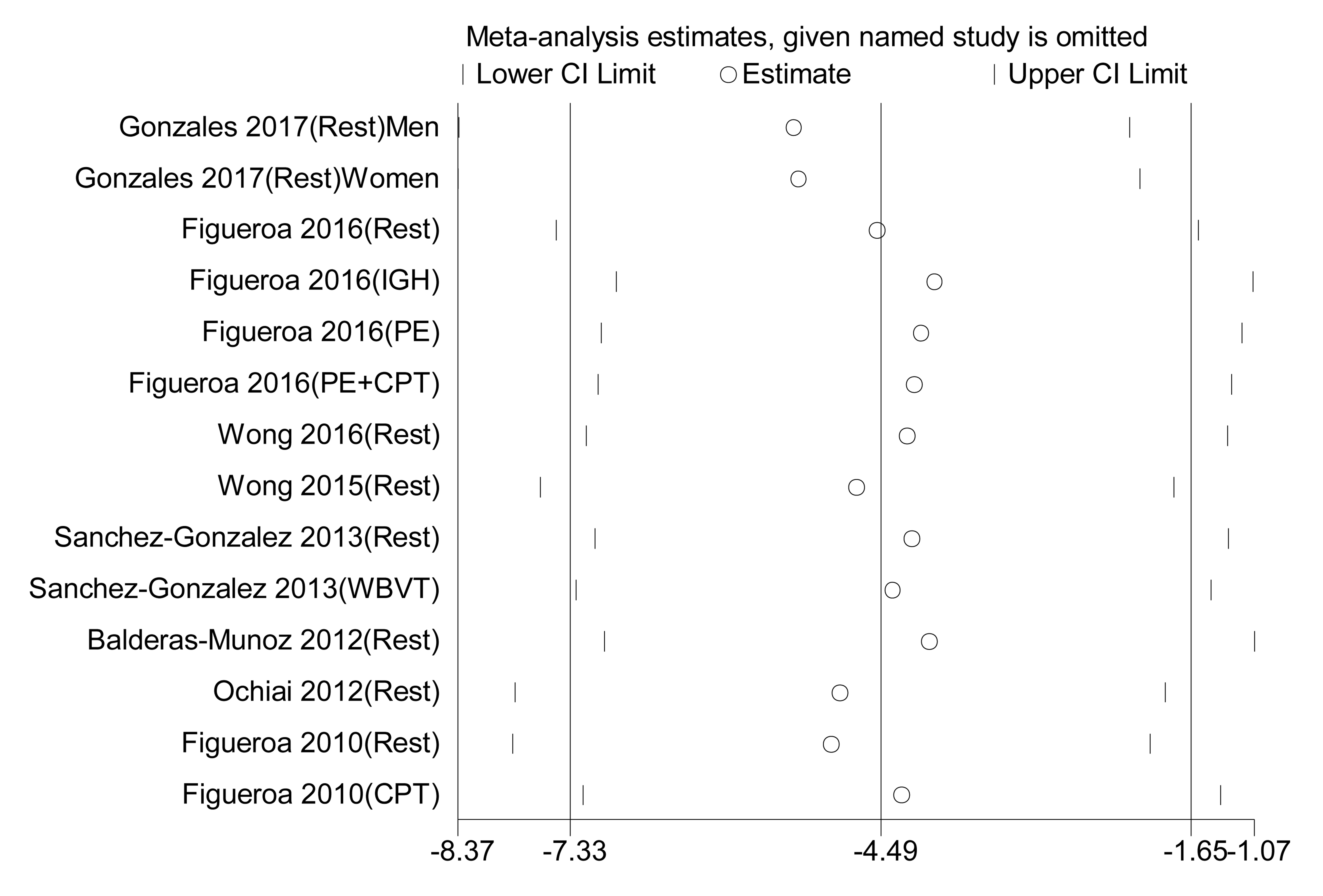

Supplement: Supplementary file 13 — Additional file 13: Figure S13. Sensitivity analysis of the effect of L-Citrulline on brachial systolic blood pressure. Abbreviations: CPT, cold pressure test; IHG, isometric handgrip; PE, post-exercise muscle ischemia (metaboreflex); WBVT, whole-body vibration training. [file 12986_2019_415_MOESM13_ESM.tif]

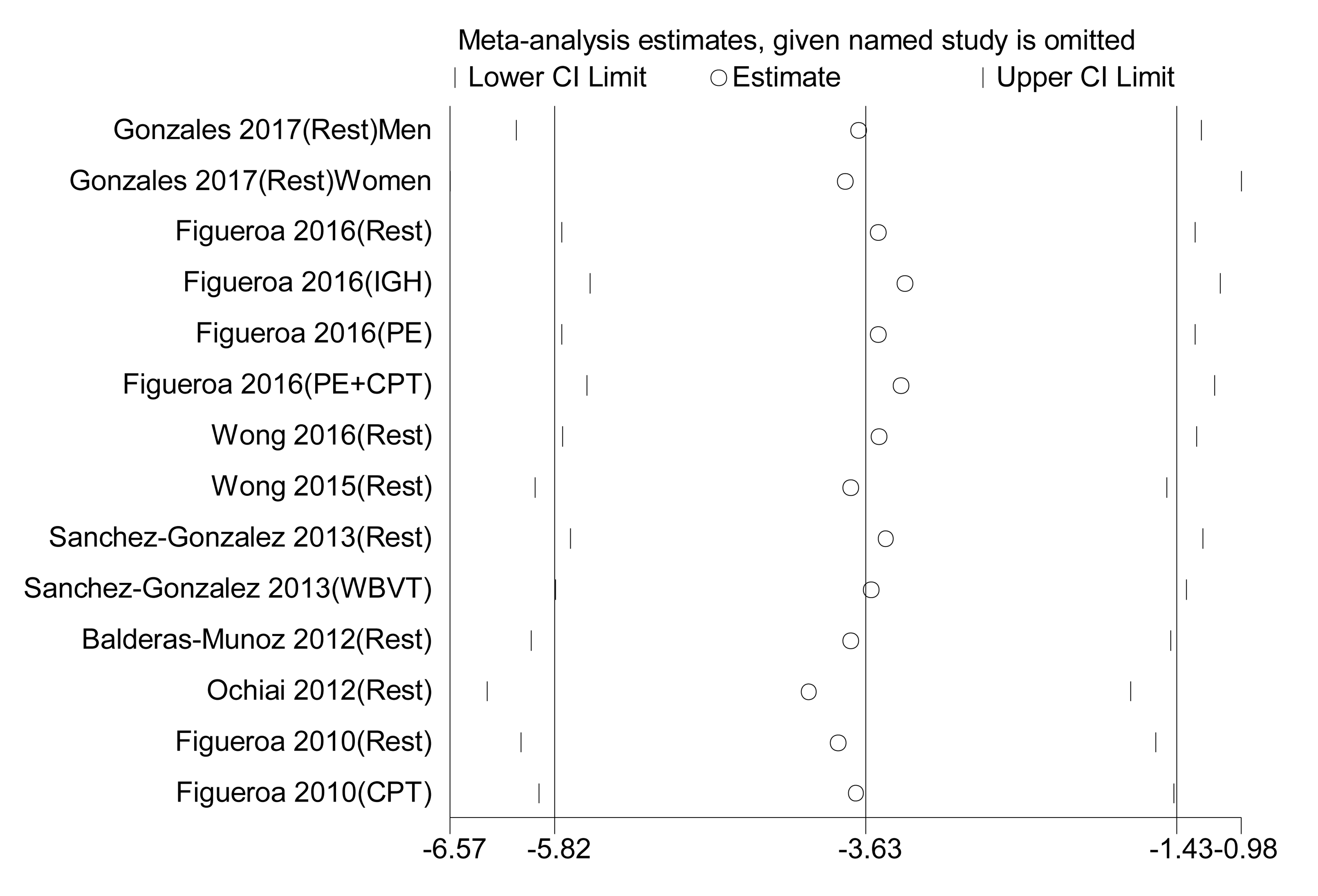

Supplement: Supplementary file 14 — Additional file 14: Figure S14. Sensitivity analysis of the effect of L-Citrulline on brachial diastolic blood pressure. Abbreviations: CPT, cold pressure test; IHG, isometric handgrip; PE, post-exercise muscle ischemia (metaboreflex); WBVT, whole-body vibration training. [file 12986_2019_415_MOESM14_ESM.tif]

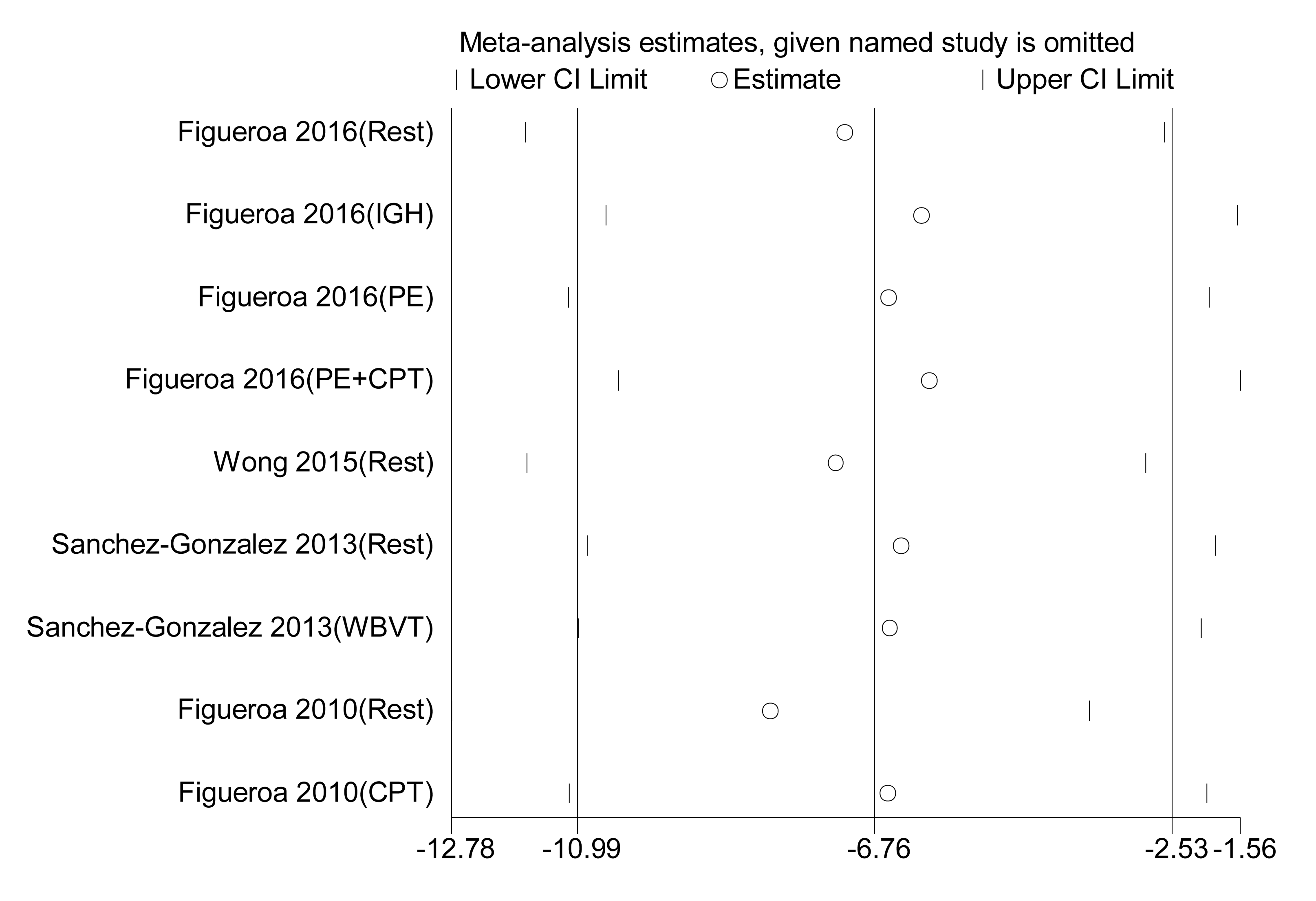

Supplement: Supplementary file 15 — Additional file 15: Figure S15. Sensitivity analysis of the effect of L-Citrulline on aortic systolic blood pressure. Abbreviations: CPT, cold pressure test; IHG, isometric handgrip; PE, post-exercise muscle ischemia (metaboreflex); WBVT, whole-body vibration training. [file 12986_2019_415_MOESM15_ESM.tif]

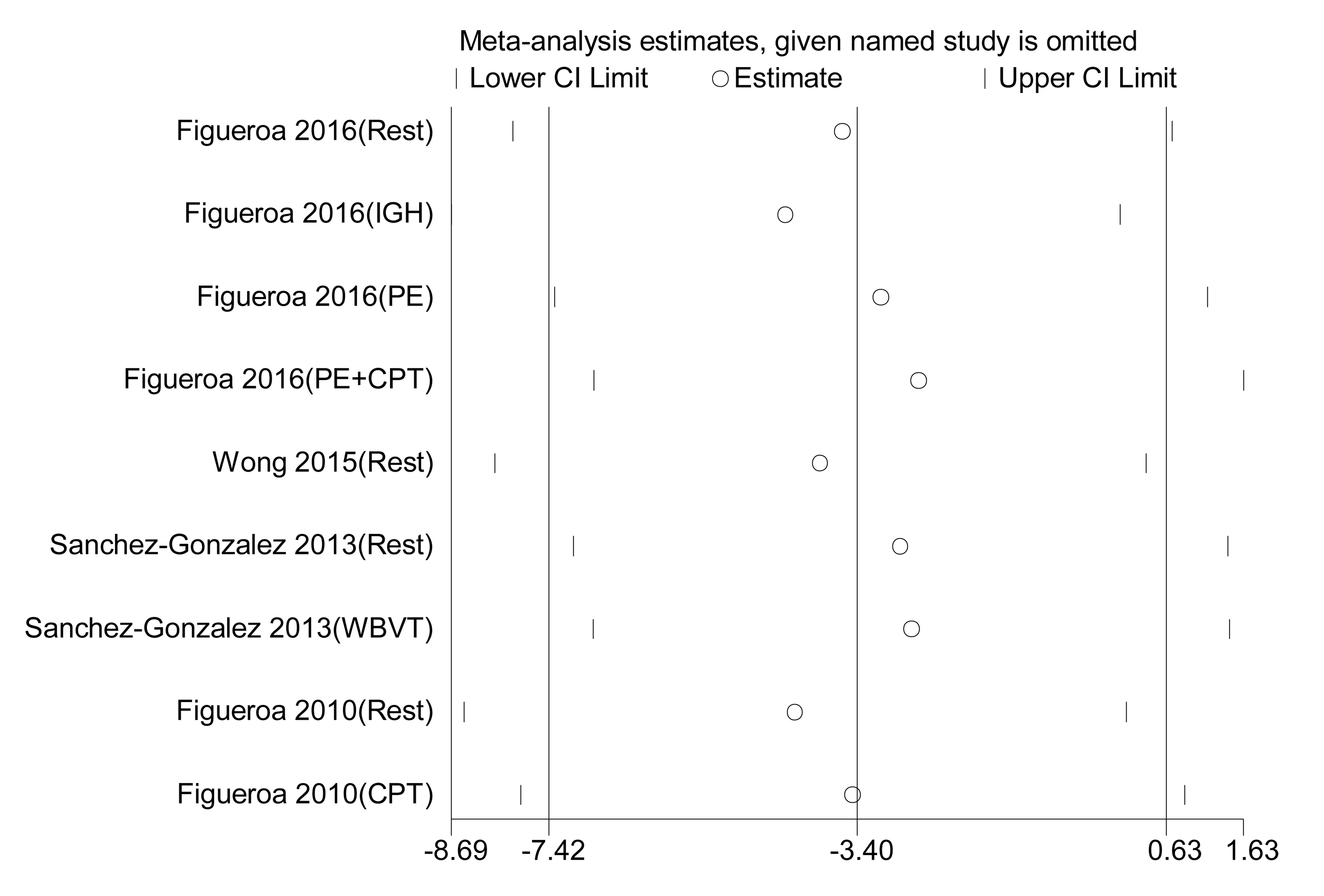

Supplement: Supplementary file 16 — Additional file 16: Figure S16. Sensitivity analysis of the effect of L-Citrulline on aortic diastolic blood pressure. Abbreviations: CPT, cold pressure test; IHG, isometric handgrip; PE, post-exercise muscle ischemia (metaboreflex); WBVT, whole-body vibration training. [file 12986_2019_415_MOESM16_ESM.tif]
