# Supplementary material for: Effect of oral L-citrulline on brachial and aortic blood pressure defined by resting status: evidence from randomized controlled trials
Source: Nutr Metab (Lond). 2019 Dec 26;16:89. doi: 10.1186/s12986-019-0415-y (PMC6933755; doi:10.1186/s12986-019-0415-y)
Supplement: Supplementary file 17 — Additional file 17: Table S1. Summarised search strategies to identify the effects of L-citrulline interventions on blood pressure. (DOCX 15 kb) [file 12986_2019_415_MOESM17_ESM.docx]

Table S. Summarised search strategies to identify the effects of L-citrulline interventions on blood pressure.

| Database | Search Strategies |
| --- | --- |
| Pubmed  Embase  Cochrane Library | ((“Citrulline” OR “L-citrulline” OR “L-Cit” OR "Watermelon") AND (“Blood Pressure” OR "BP" OR “Systolic Pressure” OR "SBP" OR “Diastolic Pressure” OR "DBP" OR “Hypertension” OR “Hypertensive”)) in All fields |
| Web of Science | TS= ((“citrulline” OR “L-citrulline” OR “L-Cit” OR "Watermelon") AND (“blood pressure” OR "BP" OR “systolic pressure” OR "SBP" OR “diastolic pressure” OR "DBP" OR “hypertension” OR “hypertensive”)) |
| Clinical Trials | Condition or disease: NA  Other terms: “Citrulline” OR “L-citrulline” OR “L-Cit” OR "Watermelon"  Study type: All Studies  Study Results: All Studies |
